# Supplementary material for: An immediate–late gene expression module decodes ERK signal duration
Source: Mol Syst Biol. 2017 May 3;13(5):928. doi: 10.15252/msb.20177554 (PMC5448165; doi:10.15252/msb.20177554)
Supplement: Supplementary file 1 — Expanded View Figures PDF [file MSB-13-928-s001.pdf]

Expanded View Figures

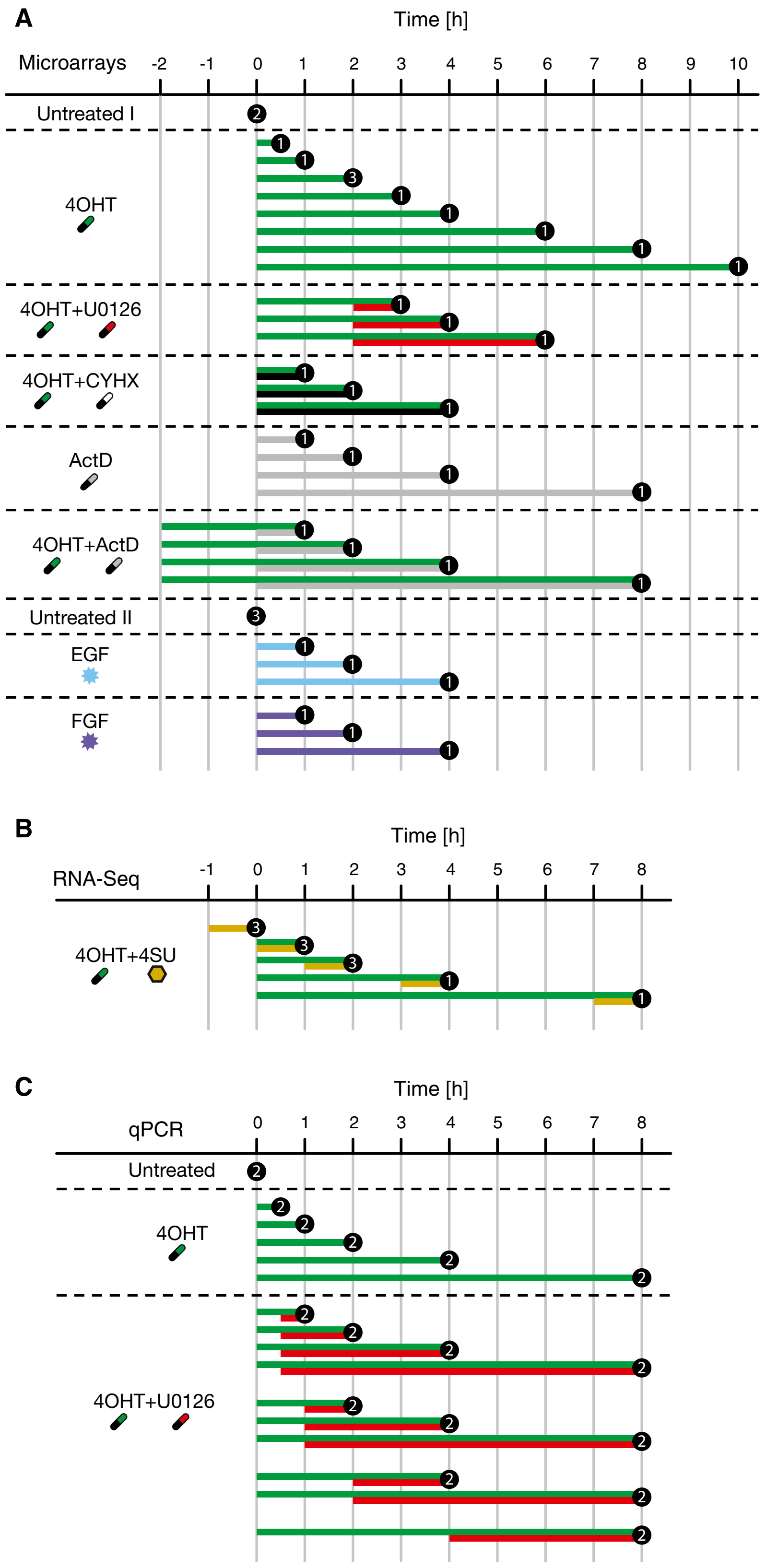

Figure EV1. Acquired samples from HEK293ΔRAF1:ER cells.

A Microarray time course data was used for model fitting and cluster definition.

B RNA-Seq time course data of metabolically labelled (4SU) cells were used for determination of transcription rates and steady-state half-lives in untreated cells.

C qPCR time course data were used for validation of the identified signal duration decoding principle.

Data information: Numbers indicate independent biological measurements per condition and time point.

© 2017 The Authors

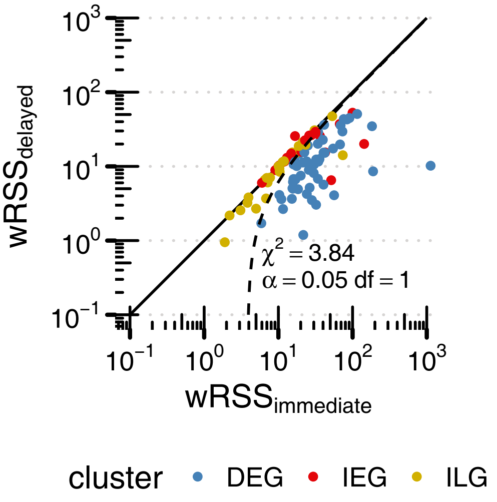

Figure EV2. Model-based temporal gene cluster identification.
Sum of weighted squared residuals (wRSS) for simple (immediate) and complete (delayed) model. The complete model was rejected for genes with $\chi^2 < 3.84$ and $\Delta t < 30$ min to only accept significantly better fitted genes for the complete model and to reflect time intervals in sampling.

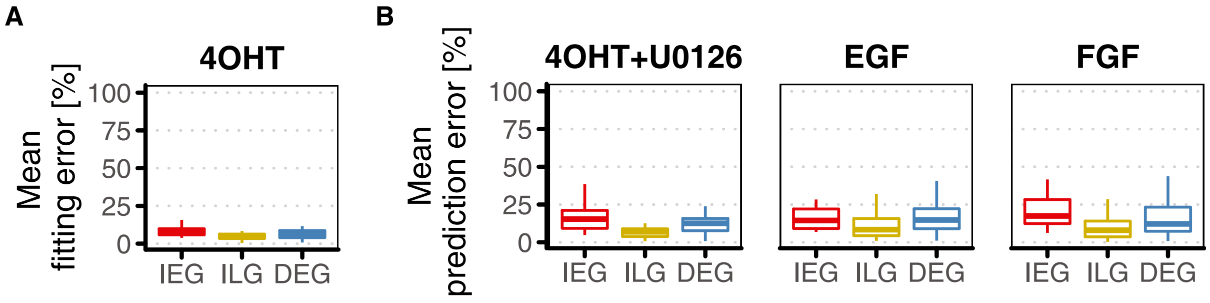

Figure EV3. Goodness of gene expression predictions.

A Mean fitting errors across temporal clusters, calculated as the mean of absolute residuals.

B Mean prediction errors across temporal clusters for tested signalling scenarios, calculated as the mean of absolute residuals.

Data information: Boxplots show median and inter-quartile range. IQR is extended with whiskers to the largest and smallest value respectively, but no further than 1.5× IQR from hinges.

© 2017 The Authors

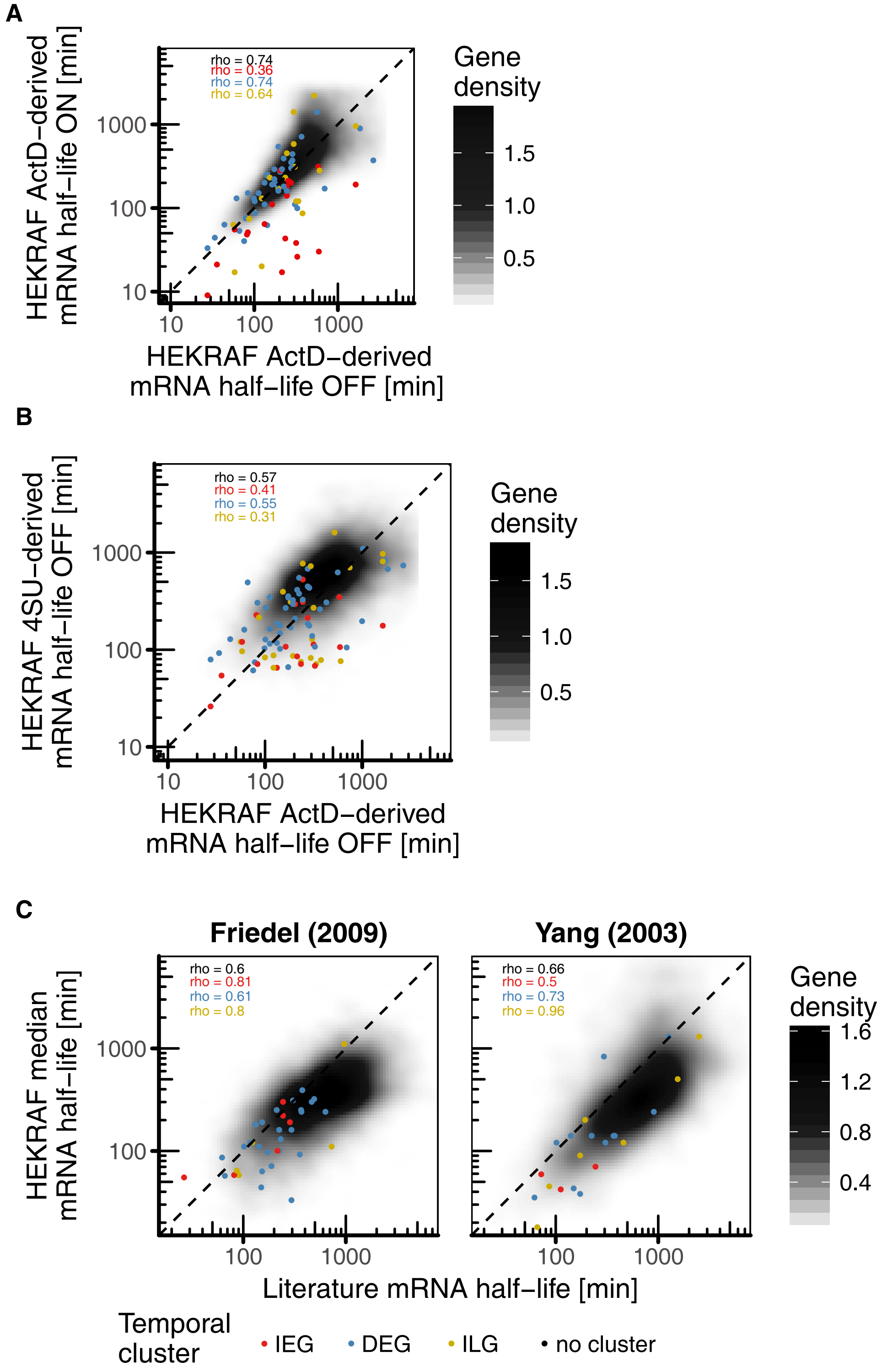

Figure EV4. mRNA half-life comparisons for HEK293ΔRAF1:ER cells.

A Treatment comparison: ActD-derived half-lives in 4OHT-pretreated versus untreated HEK293ΔRAF1:ER cells.

B Method comparison: ActD-derived half-lives compared to 4SU-derived half-lives in untreated HEK293ΔRAF1:ER cells.

C Literature comparison: HEK293ΔRAF1:ER median mRNA half-lives compared to published mRNA half-lives in two different studies.

Data information: Spearman correlation is indicated for all genes and for each gene cluster.

 © 2017 The Authors

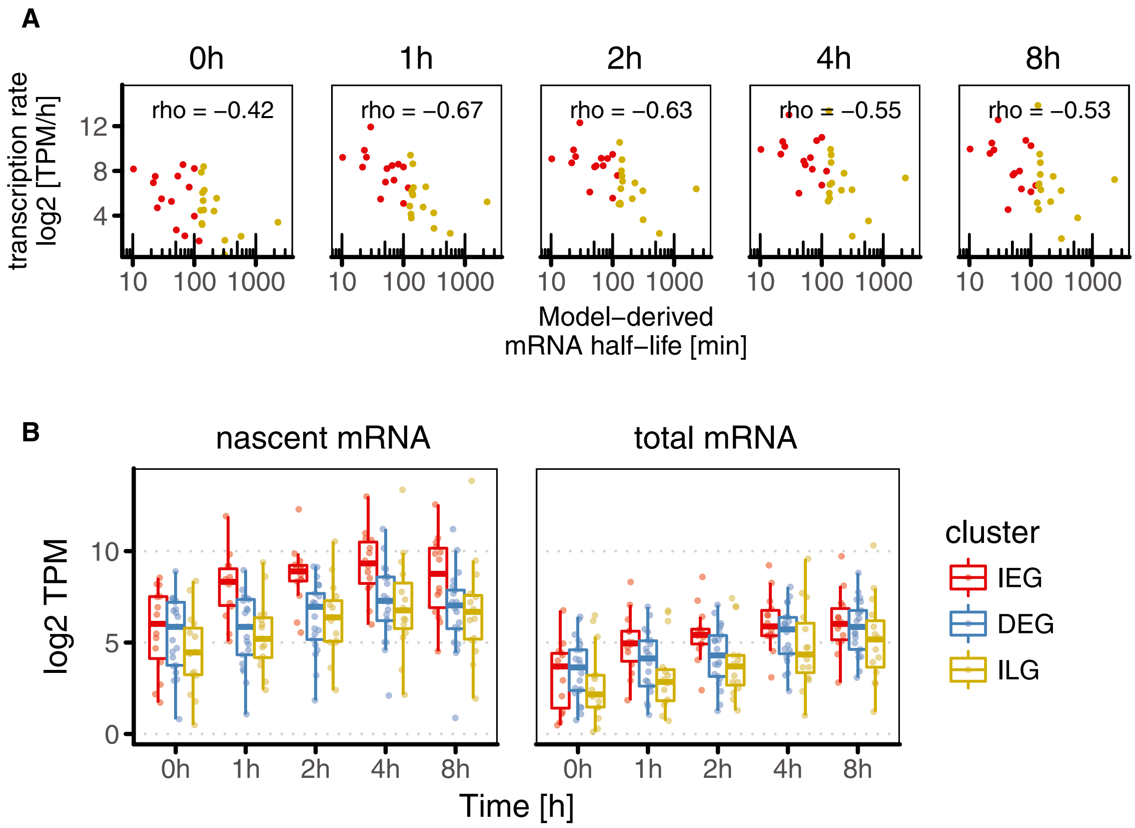

Figure EV5. Anti-correlation of transcription rate and mRNA half-life and comparison of nascent and total mRNA levels in 4OHT-stimulated HEK293ΔRAF1:ER.

A Comparison of absolute log_2 transcription rate [TPM/h = transcripts per million per hour] after different periods of 4OHT treatment and model-derived mRNA half-life in HEK293ΔRAF1:ER. Anti-correlation indicates that short mRNA half-lives in IEGs are compensated with high transcription rates.

B Comparison of nascent and total mRNA levels after different periods of 4OHT treatment in HEK293ΔRAF1:ER. IEGs have higher nascent mRNA levels after stimulation than ILGs and DEGs but end up at similar total mRNA levels after prolonged activation.

Data information: Boxplots in (B) show median and inter-quartile range. IQR is extended with whiskers to the largest and smallest value respectively, but no further than 1.5× IQR from hinges.

Figure EV6. Signal duration effects on mRNA and protein level in 4OHT-treated HEK293ΔRAF1:ER cells, and conservation of mRNA response dynamics in rat PC12 and human MCF7 cells.

A mRNA log_2 fold changes in qPCR time course data for five different ERK signal durations in HEK293ΔRAF1:ER cells (cf. Fig EV1C for treatment scheme).

B Representative Western blot for protein fold changes shown in Fig 6E. CLU and FOSL1 were measured on the same membrane; hence, lower GAPDH control corresponds to both blots.

C Comparison of HEK293ΔRAF1:ER and PC12 cells. Left panel: Spearman correlation of maximum mRNA log_2 fold changes in 4OHT-treated HEK293ΔRAF1:ER cells and corresponding homologues in NGF-treated PC12 cells. Middle panel: Spearman correlation of mRNA response times in 4OHT-treated HEK293ΔRAF1:ER cells and peak expression time points of corresponding homologues in NGF-treated rat PC12 cells. Right panel: Spearman correlation of median mRNA half-lives in HEK293ΔRAF1:ER cells and peak expression time points of corresponding homologues in NGF-treated rat PC12 cells.

D Comparison of HEK293ΔRAF1:ER and MCF7 cells.

© 2017 The Authors

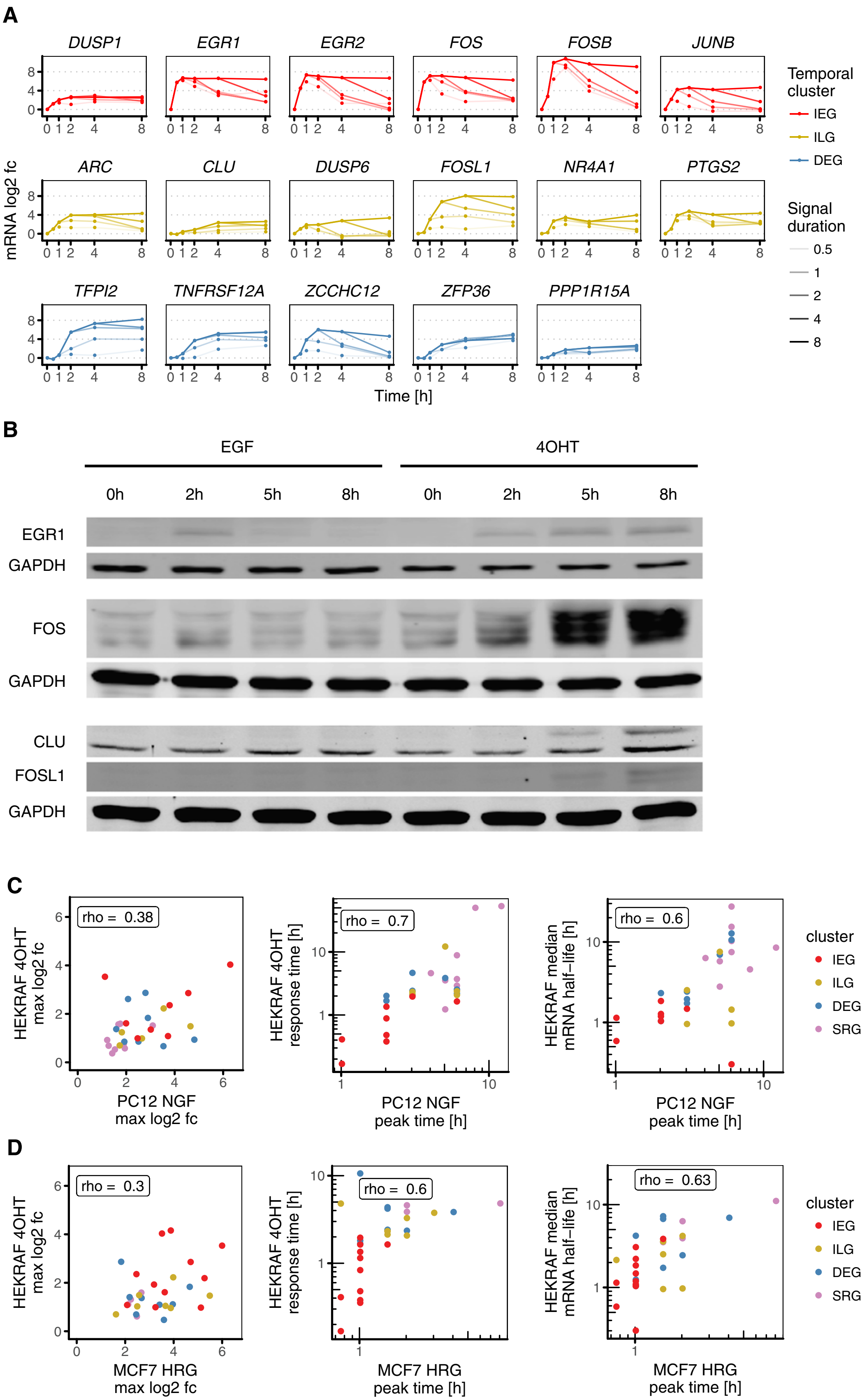

Figure EV6.

 © 2017 The Authors

A

term.name	term.id	term.size	Score	IEG	DEG	ILG
positive regulation of transcription from RNA polymerase II promoter	GO:0045944	995	3.014	9/21	2/54	5/27
positive regulation of apoptotic process	GO:0043065	569	2.772	3/21	4/54	8/27
negative regulation of protein metabolic process	GO:0051248	957	2.611	4/21	13/54	5/27
learning or memory	GO:0007611	214	2.204	5/21	0/54	3/27
positive regulation of cell proliferation	GO:0008284	814	1.618	4/21	5/54	8/27
positive regulation of biosynthetic process	GO:0009891	1749	1.59	9/21	4/54	11/27
response to lipid	GO:0033993	788	1.532	7/21	6/54	5/27
negative regulation of protein kinase activity	GO:0006469	204	1.421	2/21	6/54	1/27
negative regulation of apoptotic process	GO:0043066	844	1.338	7/21	6/54	5/27

B

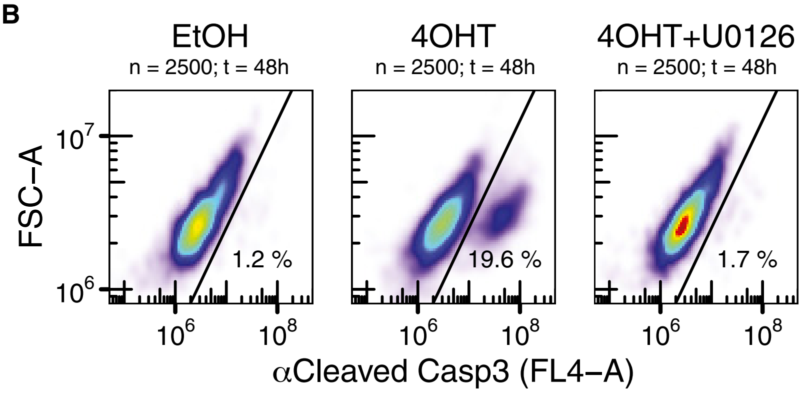

Figure EV7. Gene Ontology enrichment and apoptosis in sustained versus transiently induced HEK293ΔRAF1ER cells.

A Gene Ontology Biological Process term enrichment for IEGs, DEGs and ILGs. Score corresponds to significant enrichment in respective cluster, where Score = $-\log_{10}$ (*P*-value). Significant enrichments are highlighted with coloured border. Background colour intensities correspond to denoted fractions and are normalised column-wise.

B FACS data to detect cleaved Casp3-positive cells among untreated or treated HEK293ΔRAF1:ER cells as a marker for apoptosis. EtOH: no ERK signalling. 4OHT: sustained ERK signalling. 4OHT+U0126: 2-h pulse ERK signalling.
